# Impact of Obesity on Major Surgical Outcomes in Ovarian Cancer: A Meta-Analysis

**DOI:** 10.3389/fonc.2022.841306

**Published:** 2022-02-09

**Authors:** Benshuo Cai, Kang Li, Gang Li

**Affiliations:** ^1^ Department of Obstetrics and Gynecology, Shengjing Hospital of China Medical University, Shenyang, China; ^2^ Department of Ultrasound, Shengjing Hospital of China Medical University, Shenyang, China

**Keywords:** obesity, body mass index, ovarian cancer, surgery, complication

## Abstract

**Background:**

The impact of obesity on the surgical outcomes in patients after primary ovarian cancer surgery is unclear. We aimed at conducting a meta-analysis to evaluate the associations between obesity and major surgical outcomes in ovarian cancer patients.

**Method:**

Embase, PubMed and Web of Science databases were searched for eligible studies. Study-specific relative risks (RR) were pooled using fixed effect model when little evidence of heterogeneity was detected, otherwise random effect model was employed.

**Results:**

Twelve eligible studies were identified. The pooled incidence rates of all complications were 38% (95% CI: 29%, 47%) for obese patients and 27% (95% CI: 18%, 36%) for non-obese patients. Compared with the non-obese patients, there was a significantly increased risk of all complications in obese patients after ovarian cancer surgery, with a pooled RR of 1.75 (95% CI: 1.26, 2.43). For advanced (stages III–IV) ovarian cancer, the pooled RR of all complications was 1.55 (95% CI: 1.07, 2.24). Obese patients after ovarian cancer surgery were at higher risks of wound complication (pooled RR: 7.06, 95% CI: 3.23, 15.40) and infection (pooled RR: 1.94, 95% CI: 1.47, 2.55) compared with non-obese patients. Such increased risk was not observed for other major complications, namely, venous thromboembolism, ileus and organ failure. Hospital stay days between obese patients and non-obese patients were similar (Standardized Mean Difference: −0.28, 95% CI: −0.75, 0.19). The rates of optimal debulking (pooled RR: 0.96, 95% CI: 0.90, 1.03), readmission/return to operation room (pooled RR: 1.20, 95% CI: 0.56, 2.57) and 30-day mortality (pooled RR: 0.95, 95% CI: 0.54, 1.66) were also comparable between obese patients and non-obese patients.

**Conclusion:**

Obesity is associated with an increased risk of postoperative complications, especially wound complications and infection after primary ovarian cancer surgery. Obesity may not affect their optimal debulking rates and 30-day mortality in patients undergoing ovarian cancer surgery. Besides, to improve surgical outcomes, an advanced minimally invasive robotic approach seems to be feasible for the treatment of obese patients with ovarian cancer.

## Introduction

Ovarian cancer is the most common gynecological cancer and the leading cause of cancer-related deaths in Western countries (1, 2). Due to no specific symptoms and ineffective cancer screening, the 5-year survival remains low at less than 50% (2). Surgery is the main treatment for most ovarian cancers (1, 3). Debulking is a treatment option for ovarian cancer patients when the tumor has already spread throughout the abdomen (1, 3). Patients with optimally debulked ovarian cancer would have a better prognosis than those with sub-optimally debulked (3).

Obesity is a potential risk factor associated with higher risk of developing epithelial ovarian cancer. Obese patients are more likely to have comorbidities that may increase surgical risk compared with non-obese patients after primary ovarian cancer surgery (4). It may also affect other surgical outcomes such as optimal debulking for ovarian cancer owing to exposure difficulties (5). It is hypothesized that operating on obese patients may cause worse surgical outcomes than operating on non-obese patients (5). But so far, accumulating studies have also been evaluating the effects of obesity on surgical outcomes in ovarian cancer, but the results have been inconsistent (4, 6–16). Several studies indicated that obesity was associated with the surgical outcomes, such as optimal debulking status, postoperative complications, and return to operation room (7–11, 14, 15). But other studies did not suggest a possible link between obesity and these major surgical outcomes in ovarian cancer patients (4, 6, 13, 16).

A recent review has evaluated the association between obesity and postoperative complications after major abdominal surgery, namely, surgery on gastric, rectal, and liver cancer. However, ovarian cancer surgery was not assessed in the review (17). Only one earlier review had attempted to explore the impact of obesity on post-operative complications (4). However, due to limited studies (less than 5 studies) reviewed, the inconsistencies especially for other major surgical outcomes still have not been well addressed yet. With more evidence published recently, a comprehensive evaluation was performed to thoroughly understand the effect of obesity on post-operative complications and other surgical outcomes in patients after primary ovarian cancer surgery.

## Methods

This study followed the PRISMA checklist for guidance (18) (**Supplementary File 1**). In this meta-analysis, the study population was patients undergoing primary ovarian cancer surgery. The exposure was pre-surgical obesity. The primary outcome was all postoperative complications, and key second outcomes included other surgical outcomes such as specific complications, optimal debulking status, readmission/return to operation room, hospital stays, and 30-day mortality.

### Literature Search

Two researchers conducted the literature search independently. PubMed, Web of Science and Embase databases were searched up to Nov 2021. In the literature search, we used the Mesh terms combined with the following key words: (Obesity OR Obese OR “Body mass index” OR BMI) AND (Ovarian OR Ovary) AND (Cancer OR Tumor OR Neoplasm OR Carcinoma) AND (Surgery OR Surgical OR Operative). To avoid missing the gray literature, we searched Google Scholar as well as the reference lists of the eligible studies to identify other potential studies. More details on the search strategies are shown in **Supplementary File 2**.

### Study Selection

Eligible studies should meet the following criteria: (1) either prospective or retrospective study; (2) patients with clinically confirmed ovarian cancer as the study population; (3) preoperative obesity as exposure of interest. Obesity was defined by the WHO criteria or other regional criteria; (4) surgical outcomes as the outcome of interest such as any complications, debulking status, length of hospital stays, readmission or return to operation room, and 30-day mortality; and (5) studies should report effect estimates with 95% confidence interval or enough data to calculate the estimate. In addition, meeting abstracts, posters, editorials, or letters without full-text available were not considered in the meta-analysis.

### Data Extraction

We extracted study-specific information from the eligible studies using a structured form, which included the following domains and items: general information (authors, publication year, study design, study location, etc.), patient information (cancer diagnosis, disease status of study population, number of patients, age at diagnosis, surgical treatment, etc.), exposure information (exposure types, exposure assessment methods), outcome information (surgical outcomes, outcome ascertainment), and risk estimates.

### Statistical Analysis

To measure the association between obesity and surgical outcomes in ovarian cancer patients, we used the relative risk (RR) for categorical outcome variables and mean difference for continuous outcome variables. Heterogeneity across studies was assessed by using the I^2^ statistic. Statistical heterogeneity was considered if the I^2^ statistic was >50% (19). Fixed effects model was employed to combine the risk estimates when there was little statistical heterogeneity; otherwise, random effects model was used (20). Sensitivity analysis also was conducted for primary outcome to assess the influence of individual study on the overall result by excluding each study at a time and conducting the meta-analysis in the remaining studies repeatedly. Publication bias was evaluated by Egger’s test (21). When there was evidence of publication bias, sensitivity analysis was conducted by using the trim and fill method to adjust the publication bias (22). Statistical analyses were performed by Stata 14.0.

## Results

### Study Selection

**Figure 1:**
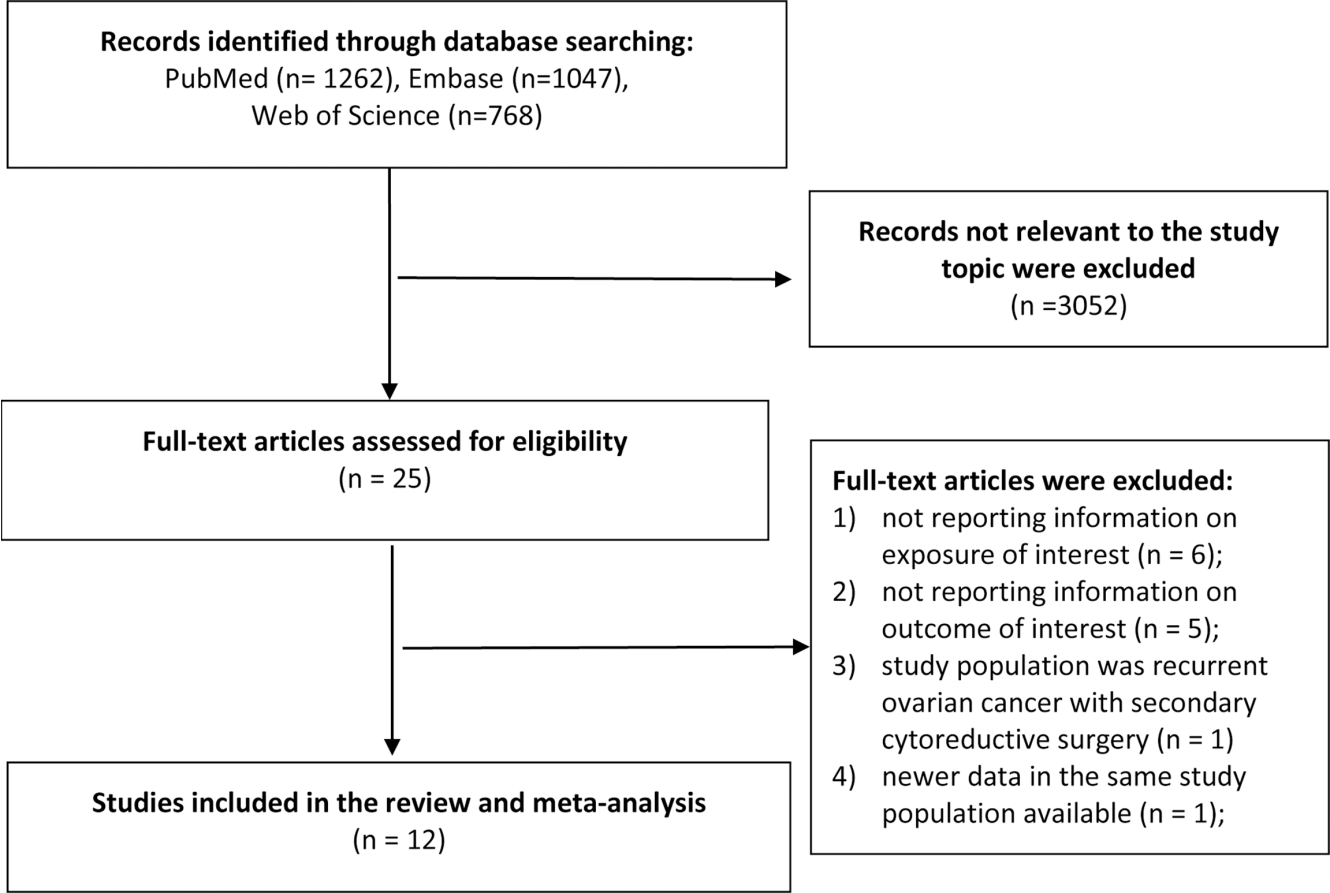
Study selection and identification in the meta-analysis.

The literature search identified 3,077 total records from the databases. After we excluded duplicates and screened the titles and abstracts, we excluded non-relevant records and identified 25 records for further full-text review. During the full-text review stage, six studies were excluded because the exposure of interest was not reported or did not meet the criteria (23–28); five studies were excluded because the outcome of interest was out of the review scope (29–33); one study was excluded because the study population was recurrent ovarian cancer with secondary cytoreductive surgery (34); and one study was excluded because of newer data available (35). Finally, we included 12 publications in the review (4, 6–16) (**Figure 1**).

### Study Characteristics

A total of 5,260 patients with ovarian cancer were included in the meta-analysis. Most of these studies were retrospective studies and only one study was a prospective study. These studies were conducted in the US (n = 5), European countries (n = 3), Asian countries (n = 2), and other countries (n = 2). Six studies included ovarian cancer patients with stages I to IV; one study included stage II to IV patients and five studies included stage III to IV patients. Nine studies defined obesity as body mass index (BMI) ≥30 kg/m^2^ based on the WHO criteria for adults; two studies in Asia population defined obesity as BMI ≥27.5 or ≥28 kg/m^2^; one study defined obesity as a visceral fat area of 100 cm^2^ or higher. Major surgical outcomes were obtained from medical record review, which included all post-operative complications, optimal debulking status, hospital stays in days, return to operation room, venous thromboembolisms, wound complication, infection, ileus and organ failure (**Table 1**).

**Table 1:** Characteristics of studies in the meta-analysis.

First author	Study design	Study location	Sample size	Tumor stage	Primary surgery	Exposure definition	Surgical outcomes
Inci et al. (8)	Prospective	Germany	106	Stage I–IV	Patients underwent maximal cytoreductive surgery	Obese defined as BMI >30 kg/m^2^	Severe postoperative complications
Heus et al. (7)	Retrospective	Netherlands	298	Stage III–IV	Patients underwent a primary or interval debulking	Obesity was defined as a visceral fat area of 100 cm^2^ or higher	All complications occurring within 30 days after surgery
Kanberg et al. (9)	Retrospective	US	507	Stage IIIC–IVB	Patients underwent primary debulking surgery	Obese defined as BMI ≥30 kg/m^2^	Any post-operative complication, infection, readmission within 30 days
Lv et al. (11)	Retrospective	China	362	Stage I–IV	Patients underwent ovarian tumor resections	Obese in Asian population defined as BMI ≥28 kg/m^2^	Complications including bleeding, infection of incision, urinary retention, intestinal obstruction, pulmonary infection, diarrhea, venous thrombosis, others
Refky et al. (14)	Retrospective	Egypt	77	Stage I–IV	Patients underwent open surgical resection that included systematic lymph node dissection (pelvic and para-aortic)	Obese defined as BMI ≥30 kg/m^2^	Postoperative complications, deep vein thrombosis/pulmonary embolism, wound complications, postoperative hospital stay in days
Castro et al. (6)	Retrospective	Brazil	83	Stage III and IV	Patients underwent primary debulking surgery or interval debulking surgery	Obese defined as BMI ≥30 kg/m^2^	30-day complications, degree of 30-day complications
Mahdi et al. (12)	Retrospective	US	2,061	Stage I–IV	Patients underwent at least a salpingo-oophorectomy, debulking, or any of surgeries	Obese defined as BMI ≥30 kg/m^2^	30-day mortality, postoperative morbidity, procedure-related complications, return to the operating room within 30 days, and length of hospital stay, 30-day complications
Smits et al. (4)	Retrospective	UK	228	Stage I–IV	Patients underwent complete and optimal cytoreduction	Obese defined as BMI ≥30 kg/m^2^ and morbidly obese defined as BMI ≥40 kg/m^2^	Surgical complications, 30-day mortality, wound complication, venous thromboembolism, ileus, return to operation room, organ failure, pneumonia, infection
Kumar et al. (10)	Retrospective	US	620	Stage IIIc–IV	Patients underwent primary debulking surgery	WHO Class III obesity defined as BMI ≥40 kg/m^2^	Surgical complications, 30-day mortality, respiratory failure, renal failure, procedure requiring anesthesia, return to operating room
Suh et al. (15)	Retrospective	South Korea	486	Stage I–IV	Patients underwent staging laparotomy for an epithelial ovarian cancer or primary peritoneal carcinoma	Obesity in Asian population was defined as BMI ≥27.5 kg/m^2^	Surgical complications, wound problem, febrile, deep vein thrombosis, ileus, hospital stay days
Matthews et al. (13)	Retrospective	US	304	Stage II–IV	Patients underwent primary cytoreductive surgery	Obese defined as BMI between 30 and 34.9 kg/m^2^, and morbid obesity was defined as BMI ≥35 kg/m^2^	Surgical complications, febrile, wound complications, deep vein thrombosis, pneumonia, myocardial infarction, transfusion, length of stay
Wolfberg et al. (16)	Retrospective	US	128	Stage III–IV	Patients underwent primary cytoreductive surgery	Obese defined as BMI ≥30 kg/m^2^	Surgical complications, hospital stay, ileus, transfusion

BMI, Body mass index.

### Meta-Analysis of Obesity and Surgical Outcomes

**Figure 2:**
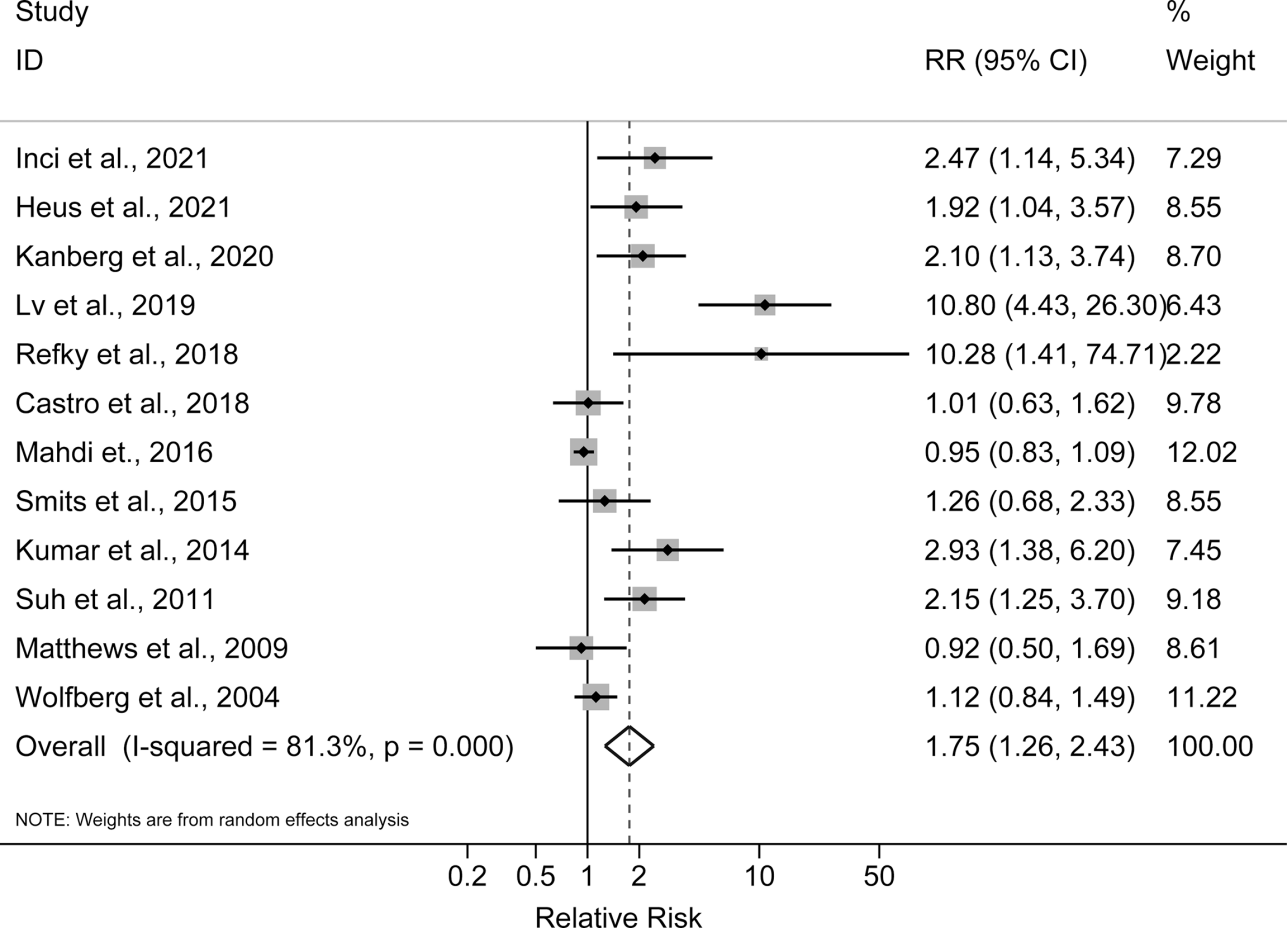
Forest plot of the association between obesity and postoperative complications in ovarian cancer patients.

The pooled incidence rates of all complications were 38% (95% CI: 29%, 47%) in obese patients and 27% (95% CI: 18%, 36%) in non-obese patients. As shown in **Figure 2**, the reported RRs ranged from 0.92 (95% CI: 0.50, 1.69) for the Matthews et al., 2009 study to 10.80 (95% CI: 4.43, 26.30) for the Lv et al., 2019 study among the 12 studies reported postoperative complications. Compared with non-obese patients after ovarian cancer surgery, the pooled RR for obese patients was 1.75 (95% CI: 1.26, 2.43), with a significant statistical heterogeneity across studies (I^2^ = 81.3%, P for heterogeneity <0.001). For advanced (stages III–IV) ovarian cancer, the pooled RR for all postoperative complications was 1.55 (95% CI: 1.07, 2.24, I^2^ = 62.0%, P for heterogeneity = 0.033). In sensitivity analysis, the pooled RRs for all postoperative complications ranged from a lowest estimate of 1.49 (95% CI: 1.13, 1.94) to a highest estimate of 1.93 (95% CI: 1.33, 2.78) after omitting the Lv et al., 2019 study and the Mahdi et al., 2016 study, respectively. Funnel plots and Egger’s test (P = 0.001), indicated potential risk of publication bias.

Wound complication (Pooled RR: 7.06, 95% CI: 3.23, 15.40; I^2^ = 0% P for heterogeneity = 0.966) and infection (Pooled RR: 1.94, 95% CI: 1.47, 2.55; I^2^ = 35.1% P for heterogeneity = 0.201) were two major postoperative complications with statistically significant higher rates in obese patients than non-obese patients. Other complications, namely, venous thromboembolism, organ failure, and ileus did not show any statistically significant excess risks for obese ovarian cancer patients compared with non-obese patients. Besides, the rate of optimal debulking (pooled RR: 0.96, 95% CI: 0.90, 1.03), readmission or return to operation room (Pooled RR: 1.20, 95% CI: 0.56, 2.57) and 30-day mortality (Pooled RR: 0.95, 95% CI: 0.54, 1.66) for obese ovarian cancer patients were comparable with non-obese patients. Hospital stay days after ovarian cancer surgery between obese patients and non-obese patients were also similar (Standardized Mean Difference: −0.28, 95% CI: −0.75, 0.19) (**Table 2**).

**Table 2:** Surgical outcomes for obese vs non-obese patients after primary ovarian cancer surgery.

Surgical Outcomes	Number of studies	I^2^ (%)	Summary Effect Estimates (95%CI)*
All complications	12	81.3	1.75 (1.26, 2.43)
Optimal debulking status	6	3.7	0.96 (0.90, 1.03)
Hospital stays in days	3	82.0	−0.28 (−0.75, 0.19)
Readmission/Return to operation room	4	68.7	1.20 (0.56, 2.57)
Venous thromboembolisms	3	0	1.29 (0.37, 4.43)
Wound complication	4	0	7.06 (3.23, 15.40)
Infection	4	35.1	1.94 (1.47, 2.55)
Ileus	3	6.2	1.01 (0.70, 1.44)
Organ failure	2	0	1.80 (0.96, 3.40)
30-day mortality	3	0	0.95 (0.54, 1.66)

*Summary estimates were pooled relative risk for categorical outcomes (i.e., optimal debulking status, readmission/return to operation room, venous thromboembolisms etc.) and standardized mean difference for continuous outcome (i.e., hospital stays).

## Discussion

There is an increasing trend of obesity incidence and prevalence worldwide (36). Obesity is a well-established risk factor for ovarian cancer, and it is estimated that obesity affects more than one-third of all ovarian cancers worldwide (37). As such, it is imperative to evaluate the impact of obesity on surgical outcomes in ovarian cancer. In this meta-analysis, we found that obesity was associated with increased risk of post-operative complications in patients after primary ovarian cancer surgery. Wound complication and infection were two major complications, which showed higher incidence rates after primary ovarian cancer surgery in obese patients than in non-obese patients. Although achieving optimal cytoreduction might be more technically challenging in obese patients; our findings indicated that obesity did not impact the ability to achieve optimal cytoreduction in women with ovarian cancer. Besides, the length of hospital stays, rate of readmission or return to operation room and 30-day mortality were similar between obese patients and non-obese patients after ovarian cancer surgery.

Whether BMI is associated with post-operative complication risk among patients undergoing major abdominal surgery for cancer is still in debate. Patients with extremely high BMI have more subcutaneous fat and thicker fat layer. Obesity may adversely affect surgical outcomes in patients after major abdominal surgery owing to limited field of view, operation difficulties during surgeries and co-morbidities (4, 38). Obese patients may also be exposed to inadequate lymph node dissection and increased intraoperative complications (4, 38). Of note, a recent review evaluated the association between obesity and postoperative complications after major abdominal surgery, namely, surgery on gastric, rectal and liver cancers. In this review, 60% of available studies found longer operative time, and 35.8% studies demonstrated a difference between obese and non-obese patients in overall morbidity of complications (38). A cross-sectional study in US women undergoing major gynecologic surgery suggested that morbid obesity (adjusted OR 1.77, 95% CI 1.45, 2.17) was associated with increased major postoperative complications after gynecologic procedures (39). Similar with the findings for other major abdominal surgery or gynecologic surgery, the current meta-analysis found a 75% higher rate of all postoperative complications for obese women than non-obese patients after primary ovarian cancer surgery. The impact of obesity was not only limited to all postoperative complications; the RISC−GYN trial also found obesity was a highly predictive factor for severe complications (8).

Substantial evidence demonstrated that obesity was associated with a number of postoperative complications, especially wound complication. Explanations include inherent anatomic features of adipose tissue, cellular and composition modifications, oxidative stress and alterations in immune mediators (40). Moreover, low blood flow in fat tissue may increase risk of infection and slow wound healing in obese patients after surgery (17, 40). Many obese patients have comorbidities such as diabetes, which also increases the risk of post-surgical infection (17). In addition, obese patients tend to sweat more, which may cause the infection of incision. Venous thromboembolism is a common complication after major oncologic surgery, and shows an estimated annual percentage increase in US (41). Although it is a leading cause of mortality of all these complications, there is no evidence of increased risk of venous thromboembolism after ovarian cancer surgery for obese patients.

The management of ovarian cancer should be personalized taking into account the performance status of the patient, in particular in the case of elderly or obese women (42). Currently, cytoreductive surgery is the most effective measure to treat ovarian tumor. Although technical difficulties may be encountered in ovarian cancer debulking, the current study did not find any significant difference in optimal debulking rates between obese patients and non-obese patients. Previous studies indicated that even morbidly obese patients with a BMI ≥40 kg/m^2^ can also achieve minimal residual disease in ovarian cancer surgery (4, 10). Obese patients can also benefit from proper treatment to manage their gynecological cancers and should not be undertreated due to the higher burden of comorbidities (42). Of note, recent advanced minimally invasive robotic approach allows a proper and safe debulking surgery for gynecologic oncologic indications, namely, cervical, endometrial, and ovarian cancers (43–45). This approach also demonstrates the feasibility, safety, and good short-term outcomes even in the very elderly and obese patients with gynecologic cancers (43–45).

There were also several limitations in the study. First, significant heterogeneity was detected, which may arise from various sources. For example, distributions of the tumor stage and the treatments of ovarian cancer may vary from study to study. For example, several studies included newly diagnosed patients with both early and advanced stages of ovarian cancer, while other studies only included patients with stage III and IV ovarian cancer. Second, most studies did not adjust important confounders such as stage, comorbidities, and surgical conditions (operative procedures, surgical time, intraoperative blood loss, etc.); residual confounding may lead to a biased pooled estimate. The potential residual confounding inherent in the original studies can hardly be addressed by a meta-analyzed approach. Lastly, publication bias may distort the association. After we adjusted the publication bias by the trim and fill method, the direction of the association did not change materially.

### Conclusion

This study suggests that obesity may raise the risk of postoperative complications in patients after primary ovarian cancer surgery. In particular, wound complication and infection should be paid more attention. However, obesity may not impact the ability to achieve optimal cytoreduction and 30-day mortality in patients undergoing ovarian cancer surgery. It is noted that obese patients can also benefit from proper treatment and additional care after ovarian cancer surgery. To improve surgical outcomes and oncological safety, a minimally invasive approach, such as robot-assisted surgery, seems to be feasible for the treatment of obese patients with ovarian cancer.

## Data Availability Statement

The datasets used and/or analyzed during the current study are available from the corresponding authors on reasonable request. Requests to access these datasets should be directed to gl-sj-cmu@outlook.com.

## Author Contributions

KL and BC developed the research design. GL interpreted the results and had primary responsibility for the final content. All authors listed have made a substantial, direct, and intellectual contribution to the work and approved it for publication.

## Conflict of Interest

The authors declare that the research was conducted in the absence of any commercial or financial relationships that could be construed as a potential conflict of interest.

## Publisher’s Note

All claims expressed in this article are solely those of the authors and do not necessarily represent those of their affiliated organizations, or those of the publisher, the editors and the reviewers. Any product that may be evaluated in this article, or claim that may be made by its manufacturer, is not guaranteed or endorsed by the publisher.

## Supplementary Material

Supplementary File 1 PRISMA checklist for the meta-analysis.

Supplementary File 2 Search strategies for the meta-analysis.
