# Supplementary material for: Impact of Obesity on Major Surgical Outcomes in Ovarian Cancer: A Meta-Analysis
Source: Front Oncol. 2022 Feb 9;12:841306. doi: 10.3389/fonc.2022.841306 (PMC8864285; doi:10.3389/fonc.2022.841306)
Supplement: Supplementary File 2 — Search strategies for the meta-analysis. [file Table_2.docx]

**Pubmed (n=1262):**

(("obeses"[All Fields] OR "obesity"[MeSH Terms] OR "obesity"[All Fields] OR "obese"[All Fields] OR "obesities"[All Fields] OR "obesity s"[All Fields] OR ("obeses"[All Fields] OR "obesity"[MeSH Terms] OR "obesity"[All Fields] OR "obese"[All Fields] OR "obesities"[All Fields] OR "obesity s"[All Fields]) OR "Body mass index"[All Fields]) AND ("ovarian"[All Fields] OR "ovarians"[All Fields] OR ("ovarial"[All Fields] OR "ovary"[MeSH Terms] OR "ovary"[All Fields] OR "ovaries"[All Fields] OR "ovary s"[All Fields])) AND ("cancer s"[All Fields] OR "cancerated"[All Fields] OR "canceration"[All Fields] OR "cancerization"[All Fields] OR "cancerized"[All Fields] OR "cancerous"[All Fields] OR "neoplasms"[MeSH Terms] OR "neoplasms"[All Fields] OR "cancer"[All Fields] OR "cancers"[All Fields] OR ("cysts"[MeSH Terms] OR "cysts"[All Fields] OR "cyst"[All Fields] OR "neurofibroma"[MeSH Terms] OR "neurofibroma"[All Fields] OR "neurofibromas"[All Fields] OR "tumor s"[All Fields] OR "tumoral"[All Fields] OR "tumorous"[All Fields] OR "tumour"[All Fields] OR "neoplasms"[MeSH Terms] OR "neoplasms"[All Fields] OR "tumor"[All Fields] OR "tumour s"[All Fields] OR "tumoural"[All Fields] OR "tumourous"[All Fields] OR "tumours"[All Fields] OR "tumors"[All Fields]) OR ("neoplasm s"[All Fields] OR "neoplasms"[MeSH Terms] OR "neoplasms"[All Fields] OR "neoplasm"[All Fields]) OR ("carcinoma"[MeSH Terms] OR "carcinoma"[All Fields] OR "carcinomas"[All Fields] OR "carcinoma s"[All Fields])) AND ("surgery"[MeSH Subheading] OR "surgery"[All Fields] OR "surgical procedures, operative"[MeSH Terms] OR ("surgical"[All Fields] AND "procedures"[All Fields] AND "operative"[All Fields]) OR "operative surgical procedures"[All Fields] OR "general surgery"[MeSH Terms] OR ("general"[All Fields] AND "surgery"[All Fields]) OR "general surgery"[All Fields] OR "surgery s"[All Fields] OR "surgerys"[All Fields] OR "surgeries"[All Fields] OR ("surgical procedures, operative"[MeSH Terms] OR ("surgical"[All Fields] AND "procedures"[All Fields] AND "operative"[All Fields]) OR "operative surgical procedures"[All Fields] OR "surgical"[All Fields] OR "surgically"[All Fields] OR "surgicals"[All Fields]) OR ("operability"[All Fields] OR "operable"[All Fields] OR "operate"[All Fields] OR "operated"[All Fields] OR "operates"[All Fields] OR "operating"[All Fields] OR "operation s"[All Fields] OR "operational"[All Fields] OR "operative"[All Fields] OR "operatively"[All Fields] OR "operatives"[All Fields] OR "operator"[All Fields] OR "operator s"[All Fields] OR "operators"[All Fields] OR "surgery"[MeSH Subheading] OR "surgery"[All Fields] OR "operations"[All Fields] OR "surgical procedures, operative"[MeSH Terms] OR ("surgical"[All Fields] AND "procedures"[All Fields] AND "operative"[All Fields]) OR "operative surgical procedures"[All Fields] OR "operation"[All Fields]))) OR (("obesity"[MeSH Terms] OR "Body mass index"[MeSH Terms]) AND "ovarian neoplasms"[MeSH Terms] AND "surgical procedures, operative"[MeSH Terms])

**Embase (n=1047)**

(obesity:ab,ti OR 'obese patient':ab,ti OR 'body mass':ab,ti) AND (ovary:ab,ti OR ovarian:ab,ti) AND ('malignant neoplasm':ab,ti OR cancer:ab,ti OR tumor:ab,ti OR carcinoma:ab,ti) AND ('surgery'/exp OR surgery OR surgical OR 'operation'/exp OR operation OR operative)

**Web of Science (n=768)**

Obesity OR Obese OR “Body mass index” (All Fields) and Ovarian OR Ovary (All Fields) and Cancer OR Tumor OR Neoplasm OR Carcinoma (All Fields) and Surgery OR Surgical OR Operative (All Fields)
